# Selections and Abstracts

**Published:** 1905-12

**Authors:** 


### SELECTIONS AND ABSTRACTS.

### FOREIGN ABSTRACTS.

BY F. G. HODGSON, M.D., ATLANTA, GA.

INFANT CONSULTATIONS.—*L'Obstetrique* (Paris) has two articles showing the excellent results of so-called "Infant Consultations" held at the Paris maternity hospitals. The mothers, being dismissed from the hospitals when the infant is two weeks old, are told to report to the hospital every week to have the baby examined and weighed to see that he is progressing in a normal manner. A careful record is kept of each baby; any abnormality in the child's condition is corrected and the mother given proper directions for his care during the following week. A short lecture is given to all the mothers on the hygiene and care of infants. Especial care is given to the care of the bottle-fed infants. The mothers take great pleasure in these meetings, where they can discuss the ills of their little ones and compare notes with other mothers. The results have surpassed all expectations, sickness among the infants is much less frequent, they are stronger and better developed and the infant mortality has been greatly reduced. These consultations are rapidly being introduced into the maternity hospitals of a number of other cities.

THERAPEUTICS OF DIRECT SUNLIGHT.—A number of observers have reported very good results from exposure to direct sunlight for an hour or more daily of such cases as lupus vulgaris, general furunculosis and chronic ulcers of various kinds. Granulating wounds and skin grafts heal more rapidly if exposed to direct sunlight daily. Cases of bone tuberculosis and tubercular glands are greatly improved if the patients have lots of sunshine. Exophthalmic goitre is often very much improved if the goitre is daily painted with a solution of potassium permanganate, rendering the skin a violet hue, and then the neck exposed to direct sunlight for an hour. The tumor may diminish in size, the nervous symptoms lessen and the pulse rate decrease. The sun's rays seem to have very much the same effect that the X-rays, Finsen rays and radium have in these cases. The sun's rays are much cheaper, are available to all and are often more beneficial than the others.

---

### ACID INTOXICATIONS AND LATE POISONOUS EFFECTS OF ANESTHETICS.

Bevan and Favill conclude an exhautive paper as follows:

From the analysis of clinical and laboratory reports bearing on this subject, we believe that we are warranted in the following conclusions.

1. Anesthetics, especially chloroform (ether to a very limited degree), can produce a destructive effect on the cells of the liver and kidneys and on the muscle cells of the heart and other muscles, resulting in fatty degeneration and necrosis, very similar to the effects produced in phosphorus poisoning.

2. The constant and most important injury done is that to the liver.

3. This injury to the liver cells is in direct proportion to the amount of the anesthetic employed, and the length of the anesthesia.

4. Certain individuals exhibit an idiosyncrasy or a susceptibility to this form of poisoning which it is difficult to explain.

5. There are certain predisposing causes which favor this destructive effect of chloroform, among which are, (a) age—the younger, the more susceptible: (b) causes which lower the general vitality of the individual and probably the vitality of the liver cells, such as diabetes, previous recent anesthesias, infections from pus germs, diphtheria, intoxications from a dead fetus in the uterus, a gangrenous mass in the abdominal cavity, etc.; (c) exhaustion due to hemorrhage; (d) exhaustion due to starvation; (e) exhaustion due to wasting diseases, such as carcinoma; (f) lesions which have resulted in extensive fatty degenerations, such as occur in the limbs in infantile paralysis; (g) chronic diseases involving both liver and kidney, such as cirrhosis and nephritis.

6. As a result of this fatty degeneration and necrosis of the liver cells, toxins are produced either from the liver cells themselves or as a result of the failure of these cells to eliminate substances which, under normal conditions, they eliminate, but which under these abnormal conditions they fail to do, and these substances, therefore, may accumulate and produce toxic effects.

7. These toxins produce a definite symptom-complex which makes its appearance from 10 to 150 hours after the anesthesia. This symptom-complex consists of vomiting, restlessness, delirium, convulsions, coma, Cheyne-Stokes respiration, cyanosis, icterus in varying degree, and usually terminates in death.

8. It is probable that milder degrees of this poisoning are recovered from, and that the transient icterus noticed after chloroform anesthesia without other evident cause is due to such poisoning, and many cases which exhibit restlessness, fright, mild delirium, drowsiness, etc., after anesthesia may be due to the same cause.

9. This disease is an hepatic toxemia; the toxins producing it hepatic toxins; and possibly the previous condition making its development easily possible should be described as liver insufficiency. Just as we have for a long time recognized a condition, uremia, in which we find arising from a variety of noxious agents, anesthetics, poisons, infections, pregnancy, etc., affecting the secreting cells of the kidney and preventing their normal function, a pathologic condition, accompanied with a certain definite symptom-complex; so we must now, we believe, recognize a condition involving the liver in which we find from a variety of noxious agents (anesthetics, poisons, infections, pregnancy, etc.), affecting the secreting cells of the liver and preventing their normal function, a pathologic condition which we must describe as hepatic toxemia, accompanied with a certain symptom-complex, and showing certain changes postmortem.

We believe that the condition of acute fatty degeneration of the liver with resulting hepatic toxemia is as definite a pathologic entity as is acute pancreatitis with fat necrosis.

10. As by-products in this toxemia, but not as the essential poisons, are found acetone, diacetic acid and betaoxybutyric acid in the blood and urine.

11. Postmortem reveals fatty degeneration of the liver, fatty degeration and mild degree of inflammation of the kidneys, and, in extreme cases, fatty degeneration of heart and other muscles. The lesion of the liver, we believe, to be the overshadowing and important one, and the one which is responsible for the symptoms and fatal result. The injury to the liver, in some cases is so great as to result in practically a total destruction of the organ.

12. Somewhat similar hepatic toxemias resulting from fatty degeneration of the liver cells occur in other conditions, and are accompanied with very similar symptoms. In such condition as phosphorus poisoning, diabetes, puerperal eclampsia, acute yellow atrophy of the liver.

13. This fatty degeneration of the liver with hepatic toxemia following anesthesia is almost invariably due to chloroform in the fatal cases. Ether is seldom the cause of a death of this kind.

14. This serious and even fatal late effect of chloroform which has heretofore not been generally recognized must still further limit the use of this powerful and dangerous agent.

15. The possibility of the development of hepatic toxemia makes chloroform distinctly contraindicated in those cases in which there exist the conditions which seem to favor its development, i. e., diabetes, sepsis, starvation, hemorrhage; the presence of intoxication from dead material ; the presence of fatty degenerations as already cited, after infantile paralysis, and lesions of the liver. The susceptibility of children to this hepatic toxemia must be recognized. That chloroform is capable of producing these serious late poisonous effects is a strong argument against its employment, and an argument in favor of the more general use of ether ; and yet we are confronted at times with the Charybdis of ether pneumonia on the one hand, and the Scilla of chloroform hepatic toxemia on the other.

16. The recognition of this danger of hepatic toxemia is a strong argument against the employment of chloroform for long anesthesia, as it can be shown that a two-hour chloroform anesthesia is almost invariably fatal to rabbits and guinea-pigs, from fatty degeneration and necrosis of the liver cells; and a two-hour chloroform anesthesia in man is an exceedingly dangerous thing.

17. These facts in regard to the late poisonous effects of anesthetics show the fact that the dangers increase with the amount of the drug employed, and with the length of the anesthesia form a strong argument in favor of rapid operating and in favor of limiting in every way possible the length of the anesthesia and the dose of the anesthetic. For example, time-consuming preparations of the patient should be made before, not after, anesthesia. In the light of this present knowledge, no surgeon can claim, as some have in the past, that after the patient is once asleep, that it makes no difference whether it requires one hour or two hours for the doing of an operation. In the light of this knowledge, for instance, three-hour breast amputations with the unnecessary ligation of fifty vessels becomes bad surgery.

18. This problem seems to us a very important one, and worthy of the most careful study and research. At present, we are practically limited to chloroform and ether as general anesthetics. Each has its danger; each has its special field in which one is safer than the other. We have, as a rule, heretofore, employed chloroform in cases in which there was a previous lung or kidney lesion, and in children, with the idea that it was less likely to produce nephritis and pneumonia, and have used ether in the bulk of our work and felt that it was specially to be selected in heart lesions. We must now add new limitations, and attempt to determine by previous examination whether there is what might be called hepatic insufficiency, the conditions present which favor the development of the late poisonous effect of chloroform on the liver. Another way of solving this problem would be the discovery of new anesthetic agents, which do not carry with them these poisonous effects, or the employment of the present anesthetic in such a way as to avoid these dangers.

For instance, it has been suggested that chloroform and oxygen combined would prevent the poisonous effects of chloroform alone, and there is some evidence to support this view. This should be determined by special research.

Again nitrous oxid and oxygen combined can be employed for long anesthesias, and experience may show that this mixture may prove to be very much safer than chloroform and ether. Although at present there is not enough evidence on which to base such a claim, in this connection I would cite the fact that the anesthetist in Dr. Bevan's surgical clinic, Dr. F. B. Moorhead, has found that after prolonged anesthesia with gas there is a marked decrease in the amount of hemoglobin, apparently a rather serious destructive effect on the blood, which may limit the use of this mixture.

In conclusion, we believe that the dangers of late chloroform poisoning must be more generally recognized and understood; that the lessons from this study are the dangers of long chloroform anesthesias and chloroform anesthesias in patients suffering from the conditions which we cited favored its development; and, we must recognize the susceptibility of children to this form of poisoning. The problem of how to prevent these deaths confront us unsolved. We have taken the first step in its solution, for we now recognize the cause of these deaths. We must work and find some way to prevent the occurrence of these serious and fatal accidents.—*Jour. A. M. A., Sept. 7th and 9th.*

---

### PRELIMINARY COMMUNICATION ON THE TREATMENT OF ARTERIOSCLEROSIS.

BY EUGEN HIRSCHFELD, M.D. (Strassburg), Honorary Physician to the Brisbane Hospital.

I wish to lay before you a short *resume* on a mode of treatment of arteriosclerosis which has given me favorable results during the last two and a half years. This method is the systematic employment of hot baths. Arteriosclerosis, we all know, is a degenerative process affecting the walls of the arteries and resulting in the impairment of their chief functions—elasticity and contractility. The causes of arteriosclerosis are mainly (*a*) advancing age: here it simply represents the sum total of labor done and injuries suffered during life; (*b*) the presence in the blood of poisonous substances, as alcohol, lead, gouty, syphilitic, malarial and other poisons, or of an excessive quantity of the ordinary waste products; (*c*) habitual over-pressure, either the result of long-continued and excessive strain, or of the irritative presence of those poisonous substances. Arteriosclerosis is the inherent penalty of "strenuous life."

No treatment whatsoever will avail against the anatomical lesion already present where fibrous growth has taken the place of the elastic and contractile elements; but we can strike at the injurious factors which, in the first instance, gave rise to the disease and are continuing to operate in the same direction. The effect of the hot bath upon the patient suffering from arteoriosclerosis is fourfold:—

1. The hot bath alters the distribution of blood pressure by unloading the internal organs and increasing the vascularization of the skin; hence it affords prompt relief in many of the various cases of pain associated with internal gout, that frequent source of arteriosclerosis. This altered distribution is also probably the reason why the sleeplessness so often troublesome in arteriosclerosis becomes after a short time manageable without the further aid of drugs. I feel sure, however, that this vascularization of the skin is not a purely derivative effect of the hot baths, but also an irritative one. It acts like a huge blister. That such is the case became evident when I used the hot bath in arteriosclerosis patients with neurasthenic symptoms. Here the abnormally enhanced sensory perception is evidently still further increased by the application of heat over the whole surface of the body, and, in consequence, the sleep is interfered with by too hot a bath. In this, as in many other cases, it was the untoward effect of the hot bath which pointed the way to its proper applicability.

2. The hot bath increases combustion. The temperature of the body rises during the hot bath. I generally had the temperature taken immediately after the hot bath and subsequently every half hour for the next four hours. This rise takes place in subjects both with a normal range of temperature and in fever cases, where the balance of temperature is disturbed. The rise depends, only to a certain extent, though, upon the warmth of the water and the length of immersion. After a bath of 104 degrees, lasting ten minutes; the rise of the body heat amounts to between one and two degrees F., and extends for about two hours; the temperature returning gradually to the former level. The rise is, of course, due not to an increased production of heat, but increased storage and direct warming of the blood. But no matter what it is caused by, the increased temperature of the body means increased metabolism, an increased oxidation of the waste products and other substances, and an increased respiratory exchange. This probably accounts, partly at least, for the loss of weight following upon the hot bath.

3. The hot bath increases the elimination of waste products. It is followed, though not immediately, by an outbreak of perspiration. If the water has not been too hot, not above 104 degrees, there is no heavy sweating, but the skin becomes moist after twenty or thirty minutes, and continues so for about one and a half hours. If the temperature of the bath is raised to 106 or 108 degrees, heavy sweating results. This sweating, no doubt, is the effort of the system to regain its former level of the temperature. It is, therefore, generally an advantage to send the patient to bed after the bath, in order to keep up a gentle perspiration for a lengthy time, although in our climate during the summer months the patient gets rather impatient of bedclothes.

4. The hot bath, by opening the channels of the skin, reduces the pressure of the blood in the same manner in which the pressure of water running from one tap is lowered as soon as another tap is turned on running. In former times it was customary to reduce the pressure by repeated venesection, nature showing the way by the occasional bleeding from the nose that sometimes relieves arteriosclerosis patients. The hot bath bleeds the patient into the skin. The pulse gets softer, more frequent, and often dicrotic. While the determination of the blood to the skin lasts the low pressure continues; hence by giving the bath at bedtime, the warmth of the bed keeps the skin vessels dilated and extends the effect. How long the fall of blood pressure continues as the result of even a few hot baths became apparent when I employed them in the treatment of other diseases where the original blood pressure was not excessively high, as in arteriosclerosis. The long continuance of the low pressure was a most unwelcome symptom, which had to be treated in itself. At any rate the systematic employment of the hot bath in arteriosclerosis daily or several times in the week at last establishes an increased vascular habit of the skin, thus permanently lowering the pressure. A patient was good enough to carry out a valuable control experiment. About twelve months ago he was under treatment for well-marked arteriosclerosis, dyspnea on slight exertion, systolic murmur over aorta, full bounding and hard pulse, increased heart dullness and polyuria; he was not able to walk 100 yards without stopping for breath. He had been under treatment for several years, had taken a trip home, and consulted various authorities there. Under careful regimen and hot baths he improved rapidly, was able to follow his occupation ever since, and could walk for five miles without getting out of breath. He abstained from liquor fairly well until about three weeks ago, when he went on an extended drinking bout. To his and my surprise, the alcohol that had always had disastrous consequences before left the patient this time without any trouble worth speaking of. He has been continuing the hot baths and been able to enjoy a good night's rest, although in former years he had had to resort to chloral from time to time.

The hot bath ought to be prescribed with as much exactness as a dose of morphia. The hot bath is a potent remedy capable of doing much good and a great deal of harm. Even the increase of one degree brings about unpleasant effects in some cases. In every instance it is necessary to ascertain exactly the reaction of the individual. One may safely begin with a temperature of 102 degrees if the patient is not above 55 or 60; in women it is safer to begin at 100 degrees. The time of immersion should not be less than ten minutes, although I rarely had to exceed that time. The determining factor in the management of the patient is the condition of the left heart, the quality and loudness of the 1 and 2 aortic sound. Without a vigorous left heart the hot bath must be used with caution. This was impressed upon me whenever I had occasion to use it. I employed the hot bath in a variety of conditions in pneumonia, meningitis, dengue, typhoid, nephritis, and the treatment of heart diseases. The most favorable results were obtained in pneumonia and dengue. But I must warn most distinctly against the indiscriminate use of the hot bath where there is not a good systematic pressure and a vigorous systolic output to start with. While these two conditions obtain in arteriosclerosis favorable results will follow the treatment indicated. I frequently, as many others before me, have used hot baths in chronic-interstitial nephritis with its high tention pulse, and have found it to fail.

The explanationin,' the light of the experience gathered, seems to me to be this: The patient generally comes under our treatment, not on account of his kidney trouble, but just at the time when the compensatory effort of the heart, maintained for years, begins to fail. As long as the heart compensates the shrinking filtering area of the kidneys by increased driving force, the patient does not become aware of his trouble. At last the heart begins to fail under the strain of the high pressure and the poison, and we have to face a failing heart, although the pressure compared with normal conditions may be high yet. In these cases the hot bath, just as well as all the sweating procedures usually recommended, does direct harm by still further lowering the needful pressure.

The following statement requires some qualifications, but, I think, covers many cases: Where digitalis is contraindicated the hot bath is likely to do good; where digitalis is indicated the hot bath should be given with caution or be left alone.—*Australasian Medical Gazette, July 20, '05.*

---

### SURGICAL SUGGESTIONS.

A very simple method of curing a corn is to excise it.—*American Journal of Surgery.*

Never incise a swelling in the course of a large artery without making sure first that it is not an aneurism.—*American Journal of Surgery.*

In wounds made by coal on the exposed parts of the body, remove all the particles of coal dust; otherwise a disfiguring pigmentation might follow.—*American Journal of Surgery.*

In case of a fresh traumatic amputation of a part of the finger, if the amputated part has not been too lacerated or crushed, try to restore the member by cleansing the parts carefully and suturing it to the stump. Once in awhile the graft will "take."—*American Journal of Surgery.*

The best drainage should be afforded for all punctured wounds of the palm; suppurations in this region are very disagreeable and are followed by severe consequences.—*American Journal of Surgery.*

After major amputations an elastic constrictor should always be left at the head of the bed, so that the nurse can immediately apply it in case of secondary hemorrhage.—*American Journal of Surgery.*

In performing subcutaneous infusion do not allow too much fluid to accumulate at one area, otherwise necrosis may occur. Shift the needle to various parts, *not* by swinging it from side to side, but by partly withdrawing it and reinserting it to another area.—*American Journal of Surgery.*

In operations upon the head or neck the anesthetist must see to it that no instrument is allowed to lie over the cornea, especially if it is exposed. Ulceration may be caused with ease; it is often healed with difficulty.—*American Journal of Surgery.*

A urethral endoscope will be found a great help as a means of introducing a rubber drainage tube into a narrow, tortuous sinus.—*American Journal of Surgery.*

If possible, always tie each component of a kidney pedicle separately, not *en masse.*—*American Journal of Surgery.*

If pus persists in the urine after an extirpation of a kidney for a suppurative disease, it often means that the ureter is involved and will require subsequent extirpation.—*American Journal of Surgery.*

In cases of suspected rupture of the bladder, catheterization is not always a sure test. The rent may be so large that the catheter draws away urine that has already flowed into the peritoneal cavity.—*American Journal of Surgery.*

Never attempt to pack a bladder for hemorrhage without the aid of guy sutures; with them one can make absolutely sure that the gauze goes *into* the bladder, and not on top of it, pushing the organ away from the space of Retzius.—*American Journal of Surgery.*

To prevent a suprapubic or other drainage tube from becoming displaced is easily accomplished by fitting another tube over it like a collar; this outer tube is split through half its length and the two portions are spread out over the skin and fastened down with adhesive plaster.—*American Journal of Surgery.*

After the open operation for varicocele the scrotum may be shortened by simply sewing the wound together transversely instead of longitudinally.—*American Journal of Surgery.*

A point worth remembering in the diagnosis of nephrolithiasis is that red blood cells are almost always found in the centrifugalized sediment in the urine even in the interval between attacks of colic.—*American Journal of Surgery.*

In performing paracentesis in the median line for abdominal fluid, be sure that the bladder is empty. When it is necessary to perform paracentesis in the lateral part of the abdomen, be careful to avoid the deep epigastric artery.—*American Journal of Surgery.*

In aspirating the chest, see to it that the syringe is in good condition before inserting the needle. Never apply the syringe to the needle after the latter has already been inserted; a severe pneumothorax may result. If the syringe is found to be out of order after the aspiration has been done, withdraw the needle also and reinsert.—*American Journal of Surgery.*

Very extensive and rapidly spreading subcutaneous infections may result after an aspiration ot a foul-smelling empyema. It is therefore wise to always operate over the site of aspiration, and especially to see that the puncture wound is well drained.—*American Journal of Surgery.*

If a patient begins to vomit long after a radical operation for carcinoma of the stomach, do not jump to the conclusion that the cause is a local recurrence. It may be a metastasis in the brain.—*American Journal of Surgery.*

---

### THE VAPOR METHOD OF ANESTHESIA.

J. T. Gwathney (*Medical Record,* October 14, 1905,) discusses the evolution in the administration of anesthetics from the time when chloroform was given "powerfully and speedily" and when an unmeasured quantity of ether was poured into the open cone up to the present, when each drop of these powerful drugs is both measured and timed. Snow, Clover, Paul Bert, Junker followed in succession and assisted in eliminating the unknown and placing anesthetics on a firm and solid basis. The Harcourt Chloroform Inhaler in England, the Braum Chloroform-Ether Inhaler in Germany, and the Gigliementi Oxygen-Chloroform Inhaler in France represent the very latest contributions towards the accurate administration of anesthetics. The objection to the French and English inhalers is, that they are for chlorofoam alone and use closed masks with valves. The Braum inhaler is the best, but the mask was undesirable. Dr. Gwathney then exhibited his own inhaler, the unique features of which are that chloroform or ether can be given singly or combined in any desired proportion; the ability to increase or decrease the air or oxygen without, at the same time, increasing or decreasing the anesthetic; the mask, an anatomically correct-fitting face piece, the rim of which is hollow and perforated around the inner margin to allow the vapor to escape, otherwise identical with a folding Esmarch mask. This is covered with four layers of gauze over which is placed a piece of oiled silk or rubber tissue. A small opening is cut in the middle of this gauze, so that, during the induction period, a few drops of chloroform may be added as with vigorous alcoholics. Dr. Gwathney's inhaler gives a maximum 2 per cent. chloroform vapor with a minimum of 1-10 per cent.

The inhaler, which is made by The Kny-Scheerer Company, consists of three ounce bottles in each of which are four tubes, varying in length from one that reaches the bottom of the bottle to one that penetrates only the stopper. These tubes represent four degrees of vapor strength. The longest, with the mask just described, has an estimated 1 per cent vapor strength; the shortest, No. 1, representing a very attenuated vapor, 1-10 per cent. As the mask is not air-tight, the vapor can not be compressed, thus avoiding the danger of an overdose. The advantages of this form of anesthesia are;

1. A pleasant induction stage.

2. Stage of excitement absent.

3. Pulse and respiration normal. No mucus râle or billowy breathing.

4. Complete relaxation.

5. Absence of unpleasant after-effects on account of the attenuated vapor used.

6. The continued use of an attenuated oxygen or air and chloroform vapor of known percentage, to which an attenuated ether vapor can be added or substituted, when conditions require a change.

7. A possible change in the vapor percentage with the same flow of oxygen or air by a change of tubes or by varying the pressure in the same tube, or by a combination of the two methods.

---

### TWO AMAZING STORIES ABOUT LIQUOZONE.

Editorial from *Truth*, of London, England, August 17, 1905.

### LIQUOZONE.

With a view, apparently, of meeting the disclosures of a recent inquest in London, the British Liquozone Company are now distributing a new batch of testimonials to the virtues of their goods. One of these is entitled "An Opinion of Liquozone by W. Gordon Stables, M.D., C.M. (Master in Surgery), Surgeon to the Royal Navy." Here are a few samples of W. Gordon Stables' opinion:

"Modern chemistry is every week playing into the hands of our profession, and we doctors are becoming more and more broad-minded. * * * We do not turn up our noses at every patent medicine we see on a druggist's counter, and some of these suit us admirably and come in most handy.

I, and many hundreds of doctors know all about Liquozone and what it can do, and has done, and will do. No secret is made of the formula. Its virtues are derived solely from the gases of the best producers of oxygen, such as manganese, dioxide, etc., etc. Its potent effect as a tonic is readily appreciated by medical men who have had clinical experience of its action. This is in itself enough, methinks, to give any invalid hope—a hope that is all but sure to be realized."

I do not think I can offer a better comment upon this valuable testimonial than the following extract from the *British Medical Journal* of July 29th:

"We do not know what scientific weight may attach to the investigations upon which Dr. Gordon Stables claims to express his opinion, but some of our readers may remember that in 1900 an advertising prospectus then issued by him fell into our hands, in the letter covering which, after asking for an advertisement for his forthcoming book, he said:

'Instead, however, of having a page advertisement at £10 10s., I should advise you to let me give you a page notice in the body of the work itself. This I will only charge £5 5s. for;' while in the enclosed prospectus he stated: 'A testimonial will, as usual, be appended to each advertisement, and the doctor will make a point of frequently alluding to these in print, and in many other ways. If desired he will send an excellent brief recommendatory letter on your specialty.'

Dr. Stables is registered as M.D., Mast. Surg., 1862, Univ. Aberd.; he entered the Royal Navy, and was placed on the retired list on June 9, 1871. We do not think this justifies him in signing himself as 'Surgeon Royal Navy,' without adding the word 'retired.'"

To this it may be added that Dr. Gordon Stables is best known as a writer of children's books. He appears to live by literary work in this shape, eked out by supplying advertisements in the shape indicated above. He therefore can not be regarded as a regular medical practitioner, and it is obvious that his opinion is not of the smallest value. If it were worth anybody's while to take the matter up, it is probable that in giving this advertisement to a company of notorious quacks he would render himself liable to the forfeiture of his diploma.

Another of the new Liquozone testimonials is given by a Mr. S. Rideal, of 28 Victoria Street, S. W., who signs himself "D.Sc. Lond., F.I.C." Mr. Rideal certifies that after making a bacteriological examination of Liquozone he found that "a 5 per cent. solution kills *B typhosus*" in less than five minutes, whereas a corresponding solution of sulphuric acid required fifteen minutes for the same job. In a further testimonial Mr. Rideal gives his opinion that "Liquozone is a perfectly safe preparation for adults" in the doses which he specifies. I do not understand from Mr. Rideal's degrees that he possesses any medical qualification; and his opinion as to the "safeness" of Liquozone may be taken for what it is worth under those circumstances. This gentleman is, however, a doctor of science of London, and it seems a questionable proceeding for a gentleman holding that degree to supply to order certificates to a firm whose advertisements are a monument of quack imposture, and who, as Mr. Rideal can easily ascertain for himself, are holding this stuff out as a remedy for dozens of diseases which have no more relation to *B typhosus* than they have to the procession of the equinoxes.

---

### THE STRANGE CASE OF MR. SMITH.

Editorial from *Collier's Weekly* for October 14, 1905.

Here's a pretty story. It shows the uses to which some hospitals are put. The Suffolk Hospital, in Boston, is a so-called charity, and there are many like it. Attendance is free, but patients pay for medicine. A. C. Smith, president of the hospital and dispensary, gets free Liquozone from the company and wants more. He writes and asks for the medicine in large quantities at reduced prices. He gets it. He writes enthusiastically of its merits on Suffolk paper. The superintendent of the hospital, a physician, is asked, on our behalf, by telephone, what this means. He replies that the hospital never uses Liquozone. A man is sent to the hospital, also on our behalf, pretending to be a patient, and he is told also that they know nothing about Liquozone. We write to the superintendent, Dr. Clarke. President Smith opens the letter and answers it, heartily indorsing Liquozone, although he is not a physician, and would have no right to prescribe Liquozone or anything else in his hospital without the knowledge and consent of the medical staffs. We then make sure that a letter to the superintendent, Dr. Clarke, is put into his own hands, this letter telling him the whole story and asking for the explanation, to which we received no reply. Going into the matter a little further, we find a testimonial of Duffy's Malt Whiskey from A. C. Smith, on Suffolk Hospital paper, and another testimonial, also written on Suffolk paper, which reads: "I can personally testify to the great remedial worth of Orangeine." It looks as if Mr. Smith's activities might diminish. Liquozone, at least, has cut him off from further supplies. How many such persons are at the head of semi-public hospitals?

---

### SOME HEART POINTS FOR MEDICAL EXAMINERS.

R. Ellis (in the *N. Y. Med. Jour. and Phila. Med. Jour.*, September 2, 1905,) closes his paper on "Some Heart Points for Medical Examiners," as follows:

### COMMON MISTAKES.

1. Excited, nervous heart, beating against thin chest walls; diagnosticated as hypertrophy.

2. Loud systolic murmur, widely diffused over left chest and behind, and disappearing with the anemic condition; diagnosticated as mitral regurgitation.

3. A loud mitral systolic murmur clearly heard six months after typhoid fever, miscalled organic mitral regurgitation. In three months such a heart may regain its muscular strength, the dilated mitral orifice may contract to its normal size, and the leak prove to be only relative.

4. Flint's aortic regurgitation heard at the apex miscalled mitral stenosis, though the peripheral signs show the true lesion.

5. Forgetting to hunt for the aortic murmurs at the third and fourth left and second right interspace because the sounds of the apex are clear.

6. Diagnosticating a cardiorespiratory murmur heard about the apex, in the axilla, and behind, as mitral regurgitation.

7. A condition where the apex beat is two inches to left of nipple line, a heaving heart, a clear first sound, an intensified second, with tense arteries, miscalled a normal heart.

8. An aortic systolic murmer diagnosticated as true aortic stenosis, when there is a strong aortic second sound, no cardiac thrill, and no low plateau pulse. It is well to remember, as has been said, that there are ten possible causes for an aortic systolic murmur, one of which is aortic stenosis.

9. The greatest mistake of all and often the real cause of most mistakes: Listening to a heart through the clothing—from a thick shirt to shirt, waistcoat and coat.

GENERAL POINTS.

1. A musical ear is of great value in heart examinations.

2. Most murmurs are functional or cardiorespiratory. Advanced heart lesions may show no hypertrophy, no dilatation, but merely a diseased valve with signs of heart muscle degeneration.

3. Cardiorespiratory murmurs disappear after a full expiration. Relative murmurs from hearts weak after acute disease disappear when the heart muscle recovers its normal tone.

4. Limit all murmurs to one diseased valve, if possible, since post mortem examinations usually show one diseased valve with relative leakage at another valve.

5. All aortic regurgitation may produce four murmurs with but one diseased valve.

6. Each murmur must have its own maximum intensity and its own area of diffusion.

7. An organic murmur is not always attended by the expected changes in the heart, in the pulmonary and peripheral circulation.

8. The pulmonary second sound is usually greater than the aortic second until thirty years of age; about the same until sixty years and less aortic second after sixty years.

9. Know well the classical pulmonary, peripheral and cardiac signs of the various cardiac lesions and see how many of these signs are practically noted in each special case, remembering that no two hearts, normal or abnormal, are ever exactly alike. If the valves are in such a good condition as to require exercise to increase the force of the blood current before the murmur is heard, such condition should be mentioned on the application.

A long, loud murmur may indicate a strong heart with little valve change, while a short, blowing murmur may indicate a weak heart with great valve change. Hypertrophy may be caused by over-exercise, by pulmonary or peripheral circulation resistance, as well as by valve lesion.

The order of frequency of valvular lesions is: Mitral regurgitation, aortic regurgitation, mitral obstructive, aortic obstructive.

Severe exercise or acute disease may temporarily enlarge a cardiac orifice without impairing the valves in the least. Post-mortem records show that about one-half of all valvular lesions are recognized during life.

The tricuspid valve is very seldom diseased, though it often leaks to relieve overtension in the right ventricle. Any examiner can hear a loud murmur from a strong heart forcibly pumping blood through diseased valves, but few recognize a low murmur from a weak heart feebly pumping blood through diseased valves.

A bad heart does not necessarily mean a valvular lesion any more than valvular lesion means a bad heart.

A pulse that intermits occasionally may be considered physiological, but an irregular, intermittent pulse is almost always pathological.

First study the heart as a pump muscle, then study the valves, then the vessels, and last, the non-organic murmurs.

---

### THE PREVENTION OF THE CONSEQUENCES OF GONORRHEAL INFECTION.

It is not many years since both the medical profession and the laity were firmly of the opinion that infection by the gonococcus was a very limited condition, and aside from the local lesions which it produced was incapable of doing further damage. The rapid advances in bacteriological study, and particularly the examination of the blood in certain cases of endocarditis and arthritis, have proved that it is quite possible for the gonococcus to enter the circulation and to produce serious lesions in portions of the body far removed from the genito-urinary tract. These lesions when they occur are in the great majority of cases incurable, and some instances of what appears to be at first glance rheumatoid arthritis are in reality cases of infectious arthritis due to this microorganism.

It is important that physicians should remember these facts not only for purposes of diagnosis, but also because they may warn their patients of the fact that gonorrhea may become a systemic malady and imperil their vitality. This is also important because it causes the physician to bear in mind that gonorrhea produces grave ravages in the pelvic organs of women, and gynecologists of large experience have stated that a very large proportion of the pelvic disorders met with in married women are due to this infection. Thus, in 1901, the Section of Hygiene and Sanitary Science of the American Medical Association, in reply to a query addressed to men of experience in this country and Europe as to the proportion of pelvic inflammation due to this cause, obtained statistics which showed that about 40 per cent. of the cases had their origin in the presence of this coccus. As Clark states in the June number of *Progressive Medicine*, the mutilation, suffering, invalidism, and death caused by the ravages of pelvic inflammation need not be dwelt upon. According to some statistics 40 per cent. of married women who suffer in this way is far too low an average. Noeggerath as long ago as 1876 declared that 50 per cent. of female sterility was due to this cause. Neisser believes that 45 per cent. of the eases of sterility have this origin, and other statistics collected by gynecologists and genito-urinary surgeons of equal eminence give a like percentage. Williams insists that 73 per cent. of all abortions are due to gonorrheal endometritis. To be sure, the statistics which we have quoted have been controverted by the studies of Bumm, who showed that a considerable number of women infected by this microorganism conceive and bear children. He thinks that two-thirds of the cases of sterility depend upon faulty development, and that only a large proportion of the last third is due to acquired sterility due to gonorrhea. Clark states that it has been estimated that from 75 to 90 per cent. of the male population of large cities have had gonorrhea, and only 5 to 18 per cent. have syphilis, and that it is remarkable in view of these facts that the prevalence of this disease has not been more taken into account in its relation to marriage. Janet speaks of gonorrhea and tuberculosis as the great pests of modern times.

This subject naturally brings up the interesting one as to whether and when the gonorrheic man may marry. Findley, agreeing with Noeggerath as to frequency and incurability of gonorrhea, questions whether physicians are ever justified in indorsing a marriage when the man has had this disease. It is quite impossible for those of us who have had large experience to believe that the mere fact that a man has had gonorrhea in previous years prohibits marriage; but on the other hand, it is undoubtedly true that a gonococcic infection may remain quiescent for long periods of time and that it may then be unintentionally transmitted without the infected individual being guilty of wrong-doing. It therefore behooves the physician, when consulted in regard to this matter, to be most conservative in his advice and to have frequent examinations made of urethral secretions before giving his permission for marriage. Findley thinks he would never sanction marriage under these circumstances, but this is undoubtedly an exaggerated view, although it serves to emphasize and impress upon our minds the important facts to which we have drawn attention.

---

CLIMACTERIC HEMORRHAGE.—A. H. F. Barbour (*Journ. of Obstet. and Gyn. of Brit. Empire,* June 1905) reports an interesting case of climacteric hemorrhage due to sclerosis of the uterine vessels in a woman aged forty-six years. Hysterectomy was performed and the microscopical findings of the extirpated uterus are described in detail.

Barbour distinguishes three groups of cases that fall under this head:

(1) Cases of arteriosclerosis, usually in women advanced in life, with hemorrhages and other marked naked eye changes in the uterine wall, but no external bleeding of consequence. This condition has been termed apoplexia uteri.

(2) Cases of uterine hemorrhage, so severe as to call for hysterectomy, of which no pathological account is given, or nothing was found to account for the bleeding.

(3) Cases of severe hemorrhage calling for hysterectomy in which arteriosclerosis was the prominent lesion. While other minor changes in the mucosa are present, such as small-celled infiltration, there is no evidence of hemorrhage or infractions, such as characterize the first group.

### THE ANTISEPTIC BABE.

BY EDNA KINGSLEY WALLACE.

We can sterilize his bottles, we can boil his little mug;
We can bake his flannel bandages, and disinfect the rug
That envelops him when he partakes of medicated air,
But there's one impossibility that leaves us in despair,—
And a not unjustifiable one, you will allow—
To wit, we fear 'twould never do to sterilize the cow!

So we feed the baby Medicus's hygienic dope,
And we wash his face with germicidal antiseptic soap;
And we brush his little toofums—or the place where they will be—
With diluted glyco-thymoline, most sanitari-lee;
Then despair to see a milky effervescence supervene
On a countenance which theretofore was surgically clean.

Thus although we strive to conquer every septic circumstance,
Yet we greatly fear a ghastly alimentary mischance;
For albeit we bake and boil his things, and scrub and soak and souse,
As if in his anatomy forever keeping house,—
The recklessness with which he sucks his vagrant tiny thumb
Imperils much his precious antiseptic little tum.

We are careful of his hours, we are thoughtful of his toys,
We are mindful of his sorrows, and judicious of his joys;
We are prayerfully considerate of needful discipline,
Of our little "Mother's Handbook" and the precepts writ therein;
And we strive to render sterile all designed for mouth or tum,
But one frightful danger menaces—we can not boil his thumb!

—*Harpers' Magazine, August, 1905.*

---

### NOTE ON CHALYBEATE THERAPY.

BY WILLIAM KRAUSS, PH.G., M.D., MEMPHIS, TENN.

*Reprinted from the Charlotte Medical Journal, February, 1905.*

There have been volumes written on chalybeate therapy, and the controversies upon the absorbability of this or that form of iron have occupied the attention of clinicians for many years. Among the many preparations claiming the attention of clinicians, the one devised by Dr. Gude, chemist, of Leipzig, has received the endorsement of the ablest men on both continents for ten years. The writer has used it to the exclusion of all others, with the exception of a few disappointing experiments with others claiming equal merit, and has published two papers upon the subject.

It would seem that when one has used a certain medicament for years, and with uniformly good results, and especially if this agent definitely eliminates the shortcomings of formerly employed products of the same elements, one is apt to take its effects as a matter of course, and further discussion would seem useless, or at least superfluous. When it happens, however, as is usually the case after a certain article has had a successful career, that many similar products strive to take the place of the original, generally resulting in disappointment to the user, it becomes a duty to take stock of the evidence in the case and see how far the confidence in the one and the distrust of the other is justified.

For this reason I have had compiled from separates and reprints some of the results of a few trustworthy observers.

1. Secondary Anemia.—H. D. Peterson (*Chicago Medical Recorder*, 1896) treated many cases, reporting five in detail, and says: "Pepto-Mangan is easily absorbed by the digestive tract without any disturbance of the same, is not injurious to the teeth, and produces no constipation."

Dr. J. W. Frieser, Vienna (*Therapeutische Monatshefte*, February, 1902), reports treating many cases, among which he made detailed studies upon forty-three cases and summarizes: "It can be warmly recommended for extensive use in the treatment of anemic conditions."

Dr. Hugo Summa, St. Louis (*N. Y. Medical Journal*, 1895), recommends its use after tests in thirty-four cases, saying: "It is especially worth mentioning that no bad after-effects could be detected. In this connection I call special attention to the absence of constipation that could be traced back to the use of this preparation."

Dr. Sam'l Wolfe, Philadelphia, reports upon fifty cases observed during about four months, and concludes: "That Pepto-Mangan is a highly available preparation of iron, on account of its liquid form, pleasant to taste, non-corrosive action on the teeth and unirritating effect on the digestive organs, admitting thus of easy graduation of dose, easy administration to children and avoidance of unpleasant effects in all cases. That it is an efficient and rapid restorer of the normal quantity and quality of the blood, etc."

Dr. Fritz Euler-Rolle, Vienna (*Wiener Klin. Rundschau*, March 29, 1903), mentions fourteen cases of anemia, besides a number of cases of other diseases, in a very complete report in which he is pleased with its absorbability and (on account of the abundance of peptone it contains) its food value in delicate stomachs, and finds it free from all the objections usually urged against iron preparations, and its results prompt.

Dr. C. A. von Ramdohr (*N. Y. Medical Journal*, June 26, 1897), in connection with some gynecological cases, reports seven cases of anemia, in which there was a rapid improvement.

Dr. H. P. Loomis, in a paper before the New York Academy of Medicine (June 18, 1893), reports a number of cases, eight in detail, in whom there was a rapid increase in red cells and hemoglobin, and in most cases with no constipating effect.

Drs. Diago and Benitez, Superintendent and Chief of Laboratory, Hospital No. 1, Havana, Cuba (*Progreso Medico*, Havana, April, 1902), report six cases in detail and summarize as follows: "We may say conscientiously that it is the best remedy we know of for the purpose, and that we do not hesitate to commend it to the profession, especially our confreres in Cuba, as an iron preparation that possesses all the advantages that can be demanded of such a remedy and none of the disadvantages that are characteristic of other iron preparations. We would especially emphasize also that Pepto-Mangan (Gude) is very pleasant to the taste, and is most easily taken by patients of all ages and with the most delicate digestions."

Dr. Juan Pablo Garcia, Havana (*La Revista Medica Cubana*, August 1, 1902), says: "I have had the opportunity of testing the efficiency of this preparation of iron in a large number of cases in both hospital and private practice, and have found it the most satisfactory iron compound that has come under my notice. It is not at all constipating, its taste is not astringent, so that it lacks the great disadvantages of most other iron compounds." He says further that it causes no disturbances of digestion, and its therapeutic efficiency has been attested by the best clinicians throughout the world.

Dr. Louis J. Gravel, Physician-in-Chief to the Hotel Dieu, Montreal (*Buffalo Medical Journal,* August, 1903), prefers the term dysemia, meaning "bad blood," reports thirteen cases with blood examination, and says: "Comparing my results with Pepto-Mangan (Gude) with those obtained from other chalybeates of this class, I have been led to give it decided preference."

---

### THE PROPHYLAXIS OF LOBAR PNEUMONIA.

J. M. Anders, M.D., LL.D. (*The Medical News,* June 3, 1905), in a discussion of this subject concludes that:

The prevalence of influenza during the last fifteen years has brought about an increased receptivity for and incidence of the pneumococcus infection.

Certain degenerative lesions, especially of the cardiovascular system and the kidneys, have shown an increased incidence during the last two decades; and these are found to be associated or antecedent conditions in the majority of cases of pneumonia, hence are probably potent predisposing factors.

The indoor conditions during the cold season favor multiplication and propagation of the pneumococcus and at the same time tend to diminish bodily resistance.

The aged are peculiarly susceptible to pneumococcus infection, hence their bodies should be kept as strong and healthy as possible, especially during the pneumonia season.

To overcome the predominating factors in individual predisposition, special attention must be paid to the subject of ventilation, to appropriate clothing, and the avoidance of agencies that cause degeneration of the heart, blood-vessel system, and kidneys, as alcohol, social excesses, an overstrenuous business or professional life, and the like.

The sputum is the principal source of infection, and should be thoroughly disinfected so soon as it is expectorated, and then destroyed by burning.

A large proportion of the general populace harbors the pneumococcus in the nasopharynx, and this is especially true in families and institutions in which cases of pneumonia have occurred, hence thorough cleanliness and systematic disinfection of these chambers should be carried out during the pneumonia season.

Means to prevent dust from accumulating and its daily removal from home and the city streets are imperatively demanded.

Public health authorities should be given full executive power to carry out rules and regulations relative to pneumonia looking to the prevention of its spread, as in the case of other infectious and contagious diseases, they should also carry on a campaign of public education.

Measures of prophylaxis must accord with intelligent public opinion before they can be rendered wholly efficient either by municipal or private authority.

---

### INDICATIONS FOR THE REMOVAL OF THE PATHOLOGIC SPLEEN.

(BYRON B. DAVIS, IN *Jour. A. M. A.*, SEPT. 2d.)

The summary of the main points discussed are:

1. The more probable function of the spleen is the manufacture of red blood corpuscles, with a strong probability of the existence of an internal secretion which is of value in maintaining the proper relative proportion of the elements of the blood.

2. Splenectomy is contraindicated in leukemia, amyloid spleen, splenic hypertrophy secondary to cirrhosis of the liver, secondary malignant disease and in the essential anemias.

3. Splenectomy is usually preferable to splenopexy in wandering spleen, which is almost always due to previous hypertrophy.

4. In abscess of the spleen, if drainage can be successfully accomplished, it is preferable to splenectomy, especially if the splenic tissue is not destroyed.

5. In cysts, benign tumors, tuberculosis and sarcoma, splenectomy is the operation of choice, unless in the three former conditions resection of the lower extremity will remove all of the disease.

6. In rupture, the organ should usually be removed. The operation should be done promptly, expeditiously, and with every expedient calculated to relieve and to prevent shock.

7. In the severe type of malarial spleen, with failure of any relief of the malaria or the extreme splenic enlargement by medical means, splenectomy will often result in cure.

8. In splenic anemia, internal medication has proven futile. The only treatment that should be considered is splenectomy, which should be done at as early a period as possible while the patient is able to withstand the operation and before incurable complications have arisen.

---

### THE ANTISEPTIC TREATMENT OF THE SUMMER DIARRHEAS OF INFANTS.

Of the various agents that have been suggested for the disinfection of the intestinal tract, Acetozone is by far the most promising. It has been shown by Novy and Freer, of the University of Michigan, that Acetozone, even in weak solutions, destroys bacillus pyocyaneus, bacillus coli, bacillus typhosus, bacillus diphtheriæ, vibrio choleræ, staphylococcus pyogenes aureus, and steptococcus in less than one minute. These writers say that "while the strong solution kills everything almost instantly, the weaker solution (1:3000) destroys the vegetating germs, as a rule, within one minute." At the same time solutions of 1 to 1000 strength are given internally without the least harmful effect. The good results accruing from the use of this remedy in the summer complaints of young children are early and unmistakable; the discoloration and putridity of the stools disappear; the diarrhea is checked; the temperature falls; pain and inflammation subside; the vomiting is controlled; and the condition of anguish and irritability is consequently greatly dispelled.

In dealing with this class of cases the following make up the round of treatment: (a) withdrawal of milk and the substitution of thin broths, albumen and cereal waters, or other liquid feedings; (b) immediate evacuation of the stomach and intestines by stomach-washing and intestinal flushing with Acetozone solution (1:5000 or stronger); (c) the sustaining of the patient's vitality; (d) administration of an internal antiseptic—Acetozone (1:3000 to 10(001 (e) the observance of hygienic conditions. In giving the drug, the solution usually administered to adults (15 grains to the quart) should be diluted with one-half its quantity of water and flavored with lemon or orange juice. It should be given in teaspoonful doses at frequent intervals—every twenty or thirty minutes at the beginning, lengthening the intervals as the case progresses.

Colonic irrigation is a useful procedure in cholera infantum. Acetozone (1:5000) solution is unexcelled for this purpose. The same solution may be used for lavage, which is recommended by many leading authorities. In washing out the stomach the irrigation fluid invariably should be lukewarm and is best introduced prior to the feedings. Its continuance must be based on the character of the washings.

Acetozone is marketed in ounce, half-ounce, and quarter-ounce vials, and in boxes containing six vials of 15 grains each. An ounce is sufficient to make eight gallons of aqueous solution.

---

### HYDROPHOBIA DEVELOPING EIGHT MONTHS AFTER PRIMARY INJURY.*

BY C. H. SCHUTT, M. D., ST. LOUIS, MO.

The patient, Henry P., a school-boy, aged 9 years, and a resident of this city, was admitted to the City Hospital at 8:30 p. m., March 17, 1905. He was accompanied by his father, was able to walk and seemingly had strength and good use of his muscles, but appeared very much excited, would not remain seated in one place but a few minutes and talked only when spoken to; his answers were intelligent and showed an exceeding keenness of perception and increased mental activity. His sense of hearing was acute—he answering questions from such a distance that ordinarily they would not be heard.

His father stated that the boy was bitten near the base of his right thumb by a stray cat last July. The cat escaped. A definite account of the accident was not obtainable. The bite healed nicely, leaving a scar one inch long. Further trouble was not experienced until about a week previous to his entering the hospital. At that time, while at school, he noticed a headache, vertigo and

*Read before the Medical Society of City Hospital Alumni, April 6, 1905.

felt weak, and was allowed to go home. This condition gradually grew worse, swallowing became difficult and he retched and vomited during a period of one day before he entered the hospital.

The patient was a white boy, weighing about 85 pounds, fairly well nourished; skin pale, moist, slightly flushed over the cheeks and apparently healthy; muscles firm; eyes large, slightly exophthalmic and pupils widely dilated; teeth good; tongue clean and mucous membranes had a very deep-pink color; chest fairly well formed, respiration arythmical, breathes freely and normally three or four times then suddenly gasps and breathes rapidly and deeply several times, then respiration ceases for a short time. At other times he had attacks of dyspnea. He had no appetite and could eat but little—he ate only a quarter of an orange while in the hospital. He could not swallow any liquid and the sight of it caused an intense feeling of disgust, and at times spasms of the throat muscles; complained of soreness in the throat but no areas of inflammation could be found. The cardiac area was slightly increased, the apex beat was forceful, the sounds were not easily made out, no murmurs were heard: pulse rapid, irregular, weak and easily compressed.

He said that his bowels had not moved for ten days and a laxative enema was ineffective. His urine was passed normally. All his special senses appeared to be intensified. His temperature was 9.96°. He complained of slight drafts through the ward, saying that they made him uncomfortable. He was put to bed and remained there voluntarily. Attacks of dyspnea were frequent; he frothed at the mouth and became very restless about half an hour before his death 3:45 a. m., March 18th.

The post-mortem was held by the Coroner, and Dr. Carl Fisch, who was present, reported that the usual signs of rabies were present in the brain. There was no meningitis.—*Courier of Medicine.*

---

THAT Acetozone is a valuable germicide is demonstrated by its effects upon typhoid bacilli and cholera vibrios in river water. In their experimental work Freer and Novy (contributions *Medical Research*, page 107) made the following tests:

(a) A cylindrical glass-wool filter was prepared, and on it was placed a layer of Acetozone crystals, about 3 cm. thick. A bouillon suspension of typhoid bacilli *passed once through this filter yielded a sterile filtrate,* while control tubes gave the usual abundant growth.

(b) A liter of tap-water was sterilized by heat and, when cool, a suspension of cholera or typhoid germs was added, the experiment being repeated several times. Ten to 20 milligrams ($\frac{1}{6}$ to $\frac{1}{3}$ grain) of Acetozone was added and, after thorough shaking, portions of the liquid were taken out and planted in bouillon and agar which was plated. In each instance the cholera germs were destroyed completely in five minutes and the typhoid germs in fifteen minutes, by the extremely small quantity of Acetozone used. It should be observed that the addition of 10 mg. of Acetozone to 1 liter of water represents a solution of 1 part to 100,000. Controls gave abundant growths, the plates yielding 600,000 to 800,000 colonies.

From the above experiments the authors draw the conclusion that pathogenic organisms are destroyed by extremely small amounts of Acetozone. They also suggest the practicability of this agent for the purification of contaminated waters, especially in connection with military operations. From other experiments it was found that even sewage can be rendered almost sterile by the addition of relatively small amounts of Acetozone.

Therapeutically, Acetozone is being very widely used in the treatment of typhoid fever, intestinal diseases—notably summer diarrhea in children—in gonorrhea, suppurating wounds and infectious processes generally. It is prescribed in the saturated aqueous solution which is prepared by adding 15 grains of Acetozone to a quart of water, shaking thoroughly and setting aside for a couple of hours to hydrolize. Messrs. Parke, Davis & Co., who prepare Acetozone, are sending out printed matter to physicians, containing reports of very gratifying results from the use of this interesting compound. Any physician who has not received a brochure can obtain one on request.

---

THE formula of the Gelineau Dragees which is not a secret preparation, was so arranged that it should contain a remedy for each of the principal symptoms of the distressing affection of which we were just speaking. The formula aims both at the pathological and the symptomatic condition of epilepsy, and its cure, in all of its forms, may be attributed to the use of this remedy. It is of all preparations that which I can most conscientiously recommend. —*Dr. Rathelot.*

---

BARTOW S. WEEKS,
Attorney and Counsellor at Law,
170 Broadway, New York.

August 8, 1905.

*The Journal of the American Medical Association, Dr. George H. Simmons, Editor, 103 Dearborn St., Chicago, Ill.*

DEAR SIR: On behalf of the Etna Chemical Co., proprietors and manufacturers of "Phenalgin," I write to call your attention to an article by Dr. Frank Billings, entitled "The Secret Nostrum Evil," read before the American Medical Association at its last meeting at Portland Ore., referring to the said preparation, "Phenalgin," in a manner indicating that it was a quack remedy, and improper to be prescribed by physicians. The publication in your journal of any such reference to "Phenalgin," I have advised my client would be libelous and not privileged.

I am also advised that you intend publishing a book containing formulæ in which mention is to be made of "Phenalgin." The publication of any alleged formulæ of "Phenalgin" in said book of formulæ would be an infringement of the right of my client.

I write you, therefore, to request that, in the publication of the proceedings of the Association and in your book of formulæ, you omit entirely the name "Phenalgin," thereby avoiding the necessity for legal proceedings on this account.

Trusting to hear from you that you will refrain from the publication of any reference to "Phenalgin," as indicated, I beg to remain,

Yours truly,
(Signed) BARTOW S. WEEKS.

### HEPATIC COLIC AND GASTRIC CATARRH SUCCESSFULLY TREATED BY LAVAGE OF THE STOMACH WITH HYDROZONE.

BY FRANCIS H. WEISMANN, M.D., NEW YORK CITY.

(Published by *The St. Louis Medical and Surgical Journal,* August, 1905.)

The patient, an engineer by profession, of fair size and weight, about 45 years old, of temperate habits, nervous temperament, has been a severe sufferer of hepatic colic and catarrh of the stomach for several years. Although having fair appetite, the patient had frequent attacks of vomiting a large quantity of mucus and bile.

In addition to the above symptoms, he was troubled with periodical attacks of hepatic colics, which were so severe that I was induced to diagnose his trouble as being caused by the presence of gallstones.

None of the remedies which were prescribed previous to January, 5, 1904, seemed to have any beneficial effect, while the periodical acute attacks made their reappearance more frequently (every four or five weeks).

Having read in medical journals several clinical reports in which Hydrozone and Glycozone were highly recommended in the treatment of diseases of the alimentary canal, I concluded to prescribe Hydrozone before meals and Glycozone after meals in varying doses for about two months without any appreciable benefit. A dose of castor oil was also administered every other week, while olive oil was given at bedtime.

The patient was growing weaker quite rapidly until an acute attack of hepatic colic which occurred beginning of April, 1904, plainly showed that the above treatment was not powerful enough to subdue the cause of his trouble.

Then I persuaded him to resort to lavage of the stomach with diluted Hydrozone.

I commenced treatment on the 5th of April, 1904, with Hydrozone 100 grammes, warm water one quart; the stomach was washed every third day in April and every second day in May, when the Hydrozone was increased to 150 c. c. (about five fluidounces), and was kept at that amount throughout the treatment; during the month of June the stomach was washed out every day, July every fourth day, August and September once a week.

The improvement was noticeable already at the end of April, when the quantity of bile and mucus was much lessened. In September the benefits derived from this treatment proved conclusively that it had not been used in vain. Internal treatment was by means of Glycozone, two teaspoonfuls before and after each meal, and every three weeks a good dose of oleum ricini.

Up to date the patient has not had another attack of hepatic colic since April, 1904, while he is now enjoying good health.

The results that I have obtained in this particular case are so gratifying that I resort now exclusively to Hydrozone and Glycozone in the treatment of all cases of stomach diseases and I believe that with the exception of stomach and intestinal disorders resulting from the presence of a malignant growth, all other cases can be successfully treated as above outlined.

218 E. Seventeenth street.

---

### ECTOPIC PREGNANCY.

Based upon the observation of thirty cases, Brickner, in the *Medical News*, arrives at the following conclusions regarding the etiology, course and management of ectopic pregnancy.

1. Sterility does not necessarily precede the development of ectopic pregnancy. If it does exist, its cause is often the same as the cause of the abnormal pregnancy.

2. The main characteristic of the bleeding in ectopic gestation is its great irregularity, there being no type. As a general rule it is not profuse. It may be constant or intermittent, and its character or profuseness has no relation to the type of the lesion. A chilly feeling often accompanies the bleeding, and vomiting and nausea may accompany the first flow. The uterine flow has apparently no connection with the death of the fetus.

3. The pain in tubal pregnancy is usually localized over the site of the lesion. It has no definite character; it may be cramplike over the affected tube, it may simulate labor pains, it may be sharp and sudden, or it may be of a bearing-down nature. The pain during a tubal abortion and that concomitant with the presence of a hematosalpinx is usually cramp-like.

4. The usual symptoms of pregnancy may be present. They are frequently absent, but their absence does not militate against the possibility or probability of an ectopic pregnancy.

5. Tenderness on palpation of the mass adjacent to the uterus is of great diagnostic value when taken in connection with the history and the other pelvic findings.

6. A rise of temperature between 99° and 100° F., in the absence of signs of infection is worthy of consideration in the diagnosis.

7. The causative factors of tubal pregnancy are probably numerous. Not one element but many may bring about the connection in different instances. It is likely that atavistic tendencies, congenital or acquired anomalies, pelvic inflammations, ovarian and tubal disease, all play a rôle in individual cases; but none of these factors alone is sufficient to explain all cases.

8. We have as yet no definite data by which we can differentiate diagnostically between all the varieties of ectopic gestation. Occasionally this may be done, but it is impossible always to distinguish between an unruptured tube and a tubal mole. A hematocele and a freshly ruptured tube can almost always be differentiated from the other usual lesions.

The value of Werth's dictum to regard every unruptured tube in the light of a malignant neoplasm has not diminished with the years.

---

STEGOMYIA fasciata has produced an epidemic of yellow fever in certain sections of Louisiana and adjoining States.

Stegomyia punctata has inoculated thousands with virulent malarial germs throughout the balance of the Mississippi Valley.

Tongaline, Mellier, in one of its forms as indicated, antagonizes and destroys the effects of these parasites on account of its extraordinary eliminative action on the liver, the bowels, the kidneys and the pores, whereby the poison is promptly and thoroughly expelled.

### THE ABDOMINAL BRAIN.

Byron Robinson, in an article published by the *Medical Age* on the solar plexus, which he calls the abdominal brain, summarizes its functions and significance as follows:

The abdominal brain is a nervous center—i. e., it receives, reorganizes, and emits nerve forces.

The abdominal brain is the nervous executive for the common functions of the abdominal viscera, as rhythm, absorption and secretion.

The abdominal brain was originally in function and location a vascular brain—cerebrum vasculare. Though complicated functions have been added, yet it is still a primary vasomotor center controlling the caliber of the blood-vessels and consequently the volume of blood to viscera, which determine visceral function.

The abdominal brain is a reflex center in health and disease.

It is the major assembling center of the abdominal sympathetic.

The abdominal brain is the seat of shock. A blow or trauma on it may cause shock, collapse, or death.

It is the automatic, vegetative, the subconscious brain of physical existence. It is the center of life itself.

In the abdominal brain are repeated all the physiologic and pathologic manifestations of visceral function, rhythm, absorption, secretion, menstruation, gestation, ovulation.

The abdominal brain can live without the cranial brain (and spinal cord), for children have been born alive with no cerebrospinal axis. Children have been born alive hours after the mother was dead.

The abdominal brain may be the agent of valuable therapeutics —e. g., in postpartum hemorrhage massage of the aortic plexus will stimulate the abdominal brain to control the blood-vessels of the uterus. Massage of the aortic plexus will stimulate the abdominal brain to send blood to the viscera, enhancing rhythm, secretion, and absorption, improving constipation and increasing visceral drainage.

The abdominal brain is the primary agent of rhythmic visceral motion. A wide office of the physician is to maintain regular visceral rhythm by means of rational therapeutics, as regular habits and exercise, wholesome coarse food, ample fluids, and proper rest.

---

### PUERPERAL INFECTION.

From a consideration of six varied cases, McDonald, in the *Montreal Medical Journal*, draws the following conclusions :

Autoinfection must be considered to include not only infection from foci of bacterial disease in distant parts of the body. Pregnant women suffering from such distant infection require most watchful care. Autoinfection from the genital canal is probably more common than is generally supposed. This is indicated in a study by Bumm and Sigwart of the bacteriology of the secretions of women in the later months of pregnancy. The streptococcus was found to be present in more than thirty-eight per cent. of the cases, and they conclude that with very careful examination aerobic streptococci may be found in the secretions of at least seventy-five per cent. of women during pregnancy and the puerperium. Of the women having streptococci, 20.4 per cent. had fever.

From this fact it may be seen that the presence of pathogenic micro-organisms in the genital canal is by no means sufficient evidence upon which to base a diagnosis of puerperal infection, and even when combined with constitutional disturbances the first step only has been taken towards the proper diagnosis of the condition. The term puerperal infection should be broadened to include infection elsewhere than in the uterus, and the location and nature of such lesions should be recognized before any operative measures are undertaken. This can only be done by exact physical examination, examination of urine, blood, etc., and a proper knowledge of the varied anatomical manifestations of infection. The frequency with which pain is right-sided in hydro-nephrosis and pyelitis should be remembered in differentiating the diagnosis from appendicitis.

The utter futility, and even harmfulness, of curettage in most cases is readily seen ; and when the varied character and oftentimes widespread distribution of puerperal infection is considered, the explanation for the high mortality (over seventy per cent.) of hysterectomy in that condition is obvious.

---

### BRIGHT'S DISEASE.

A radical distinction between Bright's disease and nephritis is made by the author. Nephritis is an inflammation of the kidneys; Bright's disease is a systemic affection that usually leads to nephritis, but does not invariably do so. The sequence of events which leads to Bright's disease may be stated thus: Intestinal putrefaction, absorption of toxic substances in such quantities as to overpower the liver and thus pass through it unchanged into the general circulation, rise in blood-pressure, due to such toxins. This sooner or later leads to cardio-vascular degeneration and at times nephritis. This sequence of events is Bright's disease.

Treatment should consist in combating its development at whatever stage it is encountered. Regarding specific lines of treatment, the surgical treatment of Bright's disease is mentioned only to be condemned. Acute nephritis is properly treated by starvation, but not chronic nephritis. As the nephritis of Bright's disease is the most chronic form of nephritis, starvation is irrational. A properly mixed diet which will furnish thirty calorics per kilo of body-weight is essential. Milk diet is undesirable. It contains albumin in excess, it is deficient in iron, it floods the heart and arteries with water, it dilutes too much the digestive fluids, it is monotonous. Abundant water drinking is injurious. The chief value of "sweating" depends on its power to deplete the system of water. To sweat a patient and at the same time to give him abundant water is irrational. The withdrawal of salt from the food has much in its favor, both theoretically and practically.—A. C. Crofton (*Journal of the American Medical Association*, June 24, 1905.)

---

### THE CARABANA PRIZE.

The $50.00 prize recently offered by Mr. Geo. J. Wallau for the five reasons best defining why physicians should and do prescribe carabana has been awarded to Dr. J. L. Hatch, New York.

Dr. Hatch's reasons: 1. First and foremost because it is an ideal aperient water, comprising all the good qualities of other mineral waters without any of their objectionable features, its action being rapid but gentle, with no weakening after-effects. 2. Because it contains more grains of the anhydrous mineral salts to the pint than any other mineral water. See analysis. One litre of carabana water contains:

| | | |
|---|---|---|
| Sulphate of sodium | 100.1110 | gr. |
| Sulphite of sodium | 0.0499 | " |
| Sulphate of magnesia | 3.0711 | " |
| Chloride of sodium, 2 gr | 1.6000 | " |
| " magnesium, 2 gr | 0.4774 | " |
| " calcium, 2 gr | 0.1967 | " |
| Phosphate of sodium | 0.0210 | " |
| Alumina | 0.0005 | " |
| Anhydrous salts | 106.0826 | grams. |

3. Because, besides being cathartic, sanguifacient, antiseptic and antipyretic, it is nutrifacient, a great boon to convalescents. 4. Because it can be given to patients suffering from disease of the genito-urinary tract where flushing is desired, and other waters are contraindicated on account of the presence of ammonio-magnesian calculi. 5. Fifth, lastly and always, because it is agreeable as well as efficacious, and can be borne by the most delicate and sensitive stomachs, and also appeals to the most fastidious taste. "Qui pro sunt omnibus."

---

THE following dietary is prescribed by L. Pierce Clark, in Epilepsy:

BREAKFAST.

Fruit: California grapes, grape fruit, oranges (pulp and juice), peaches, apples, pears, plums.

Cereals: Oatmeal, Pettijohn, wheatena, cracked wheat, force, grape-nuts, rice, germea, cream of wheat.

Eggs: Soft-boiled, poached, scrambled or shirred, omelet.

Bread: Dry toast, milk toast, shredded wheat biscuit, graham bread, triscuit, gluten bread, zwieback, Huntley & Palmer's breakfast biscuit, Uneeda biscuit, French bread.

Liquids: Milk, buttermilk, peptonized milk, skimmed milk, kumyss, cereal coffee, Phillips' digestible cocoa, milk and vichy, milk and egg shake.

DINNER.

Soups: Clear, consommé, purée of vegetables, bouillon with egg, chicken and mutton broth, chestnut soup, oyster soup.

Fish or Meat: Fish (white, halibut, shad, brook trout, weakfish, red snapper), broiled, baked or boiled. Fowls should be broiled, roasted, boiled, or fricasseed. Meats (roast beef or beefsteak, roast lamb or lamb chops, boiled mutton).

Vegetables: Potatoes, parsnips, celery, tomatoes, spinach, asparagus, peas, string beans, salsify, lettuce, squash, macaroni, spaghetti, rice (prepared according to directions).

Desserts: Fresh fruits as given above, plain puddings, ice cream and water ices, cheese (cream, cottage, camembert).

SUPPER.

Breads, cereals, eggs, oysters (stew, creamed, steamed), creamed sweetbread, stewed fruits (prunes, apples, peaches, apricots, rhubarb), liquids as for breakfast.

Avoid fried foods, rich gravies, pastry, cake, candies, hot bread, all forms of alcohol, tea, coffee, pork, lobster, ham, fruits with seeds.

---

### THE TREATMENT OF INFLAMMATORY CONDITIONS OF THE THROAT BY IRRIGATION.

Edward L. Kellogg, M.D., New York, says (*New York Medical Journal*, April 22d): "During the several years of my connection with the Hospital for Scarlet Fever and Diphtheria Patients, I have received many inquiries from physicians in New York and elsewhere concerning the methods adopted there for treating throat infections in scarlet fever and diphtheria.

The statement that we use saline irrigations has been followed in many cases by a request for details. A review of the modus operandi, therefore, may not be out of place, although the course pursued is not a new one.

The usual forms of amygdalitis and pharyngitis may be treated effectively in the same way.

Normal saline is the solution ordinarily used. Occasionally, when the throat is dry and glazed, a solution of sodium bicarbonate (one tablespoonful to a pint of water) is preferred to soften tenacious mucus or start secretion.

Boric acid, potassium permanganate, and other antiseptic solutions possess no apparent advantage over the salt water, which not only has a soothing and cleansing effect, but will do no harm even if a considerable quantity is swallowed while the patient is being "broken in" to the treatment.

The solution is made as hot as it can be borne without discomfort, usually 110 degrees F. at the start, with a gradual increase to 125 degrees.

The quantity used is about two quarts; sometimes, however, it is necessary to diminish the amount until the patients learn to let the water flow out of the mouth without exhausting themselves by gagging and straining.

The severity of the infection plus the ability of the patient to stand the irrigation determine the frequency of the treatment. Severe cases as a rule are irrigated every two hours from six a.m. to midnight.

The patient, protected by a rubber sheet, is turned on one side with the cheek resting on the edge of a pus basin, and the head is lowered slightly by removing the pillow. (Infants are prepared as for intubation by wrapping them from the shoulders to the feet in a strong sheet fastened firmly at the shoulders, elbows, wrists, knees, and ankles.) A fountain of Davidson soft rubber bulb syringe is used. The straight tip of the syringe is introduced into the mouth in the median line and carried back to the base of the tongue, which is held down so as to expose the back of the throat. The solution is then directed with considerable force against the pharynx, or the part of the throat from which we wish to dislodge the membrane. When the mouth is filled, the tube is compressed with the finger, and the patient is allowed to expel the solution into the basin. This process is repeated until the treatment is finished.

The effect of the treatment described is to relieve pain, dislodge membrane, and wash away thickened mucus more thoroughly than by any other means with which I am familiar.

In bad cases, particularly those in which the nasopharynx is extensively involved, the nose is also irrigated with the same solution. A conical tip which plugs the nostril tightly is employed, or a catheter is passed through one nostril to the nasopharynx. The latter method, although slightly less efficacious, is adopted to minimize the danger of ear effection, which is considerable in all nasal irrigations.

The tips supplied with the syringes are not well adapted for the work of throat irrigation; they are too short and do not offer a sufficiently broad surface to satisfactorily depress the tongue.

On this account I have devised an irrigator tip which is now in use in the Hospital for Scarlet Fever and Diphtheria Patients, and which I have used in private practice with much satisfaction."

---

### THE TESTS FOR OCCULT BLOOD IN THE FECES AND THEIR CLINICAL SIGNIFICANCE.

J. Dutton Steele (*Penn. Med. Jour.*, February, 1905) describes this aid in diagnosis:

1. Hemorrhoids, menstruation and kindred sources of hemorrhages must be excluded.

2. It is probably better to exclude hemoglobin in the form of meat or meat juices from diet for a day or so before the test is applied.

3. The stool should be made soft with some mild saline.

4. A small portion, about 5 cc. of soft or softened stool is abstracted with 20 cc. of ether to remove the fat and fatty acids which interfere with the test. The ether is allowed to rise and is poured off. A small beaker is very convenient for the test. Two or three cc. of glacial acetic acid are added to the feces and the whole is stirred thoroughly. Then this mixture is again abstracted with 10 cc. of ether. This ethereal extract is the portion to be tested. About 2 cc. of this is taken in a test tube and two or three drops of a freshly prepared tincture of guaiac are added. Instead of the tincture a little of the resin can be added directly to the ethereal extract in which it dissolves readily. Then add twenty to thirty drops of pure hydrogen peroxid, and shake. If blood is present the ethereal extract will turn a clear blue which may appear a purplish-blue if the ethereal extract is browned, as it often is. The color fades quite rapidly. Ozonized oil of turpentine may be used instead of the hydrogen peroxid. Dr. Steele's summary:

1. In cancer of the stomach or intestines the test has shown blood in almost every stool passed.

2. In ulcer of the stomach blood is not, as a rule, found in every stool, but is irregularly present. Sometimes it may miss one or two or even more stools.

Under this division of intermittent occult bleeding comes also duodenal ulcer, benign organic pyloric stenosis and spastic pyloric stenosis.

3. Occult blood is never found in gastrites acida, anacida, or subacida, hyperacidity, hypersecretion (without ulcer), benign dilatation and neurosis.

---

### SPASTIC CONSTIPATION AND ITS TREATMENT.

A. Albu, of Berlin, in the *Medical Record*, July 1, 1905, concludes his paper as follows:

1. The use of warm or hot sitz or complete baths of fifteen to twenty minutes duration.

2. Hot compresses to the abdomen to mitigate the spasm or relieve pain.

3. Warm enemata of linseed, sesame or castor oil in a quantity of one-quarter liter every evening, with the buttocks raised or in the knee-elbow position.

4. Atropine or belladonna, the latter in the form of suppositories, two or three times daily.

5. A vegetable diet, free as possible from cellulose, consisting of wheaten bread, prunes, and cooked fruits, with soups, broths, and milk if the latter is not constipating to the individual, but maintaining a careful avoidance of alcohol, spices, cabbage, cucumbers, celery, radishes and cheese.

6. The introduction of lubricated bougies into the rectum every day, for ten to fifteen minutes, especially where spasm of the sphincters is demonstrable.

Finally, the author disapproves of long and continuous rest in bed for these patients, except when very debilitated or intensely hysterical cases, as thereby the primary atony of the intestine is favored and exercise in the fresh air is rather indicated.

---

### LOCALIZATION OF CHRONIC SUPPURATIONS OF THE URINARY TRACT.

Authur L. Chute, the *Boston Medical Journal,* August 24th, discusses the methods of determining whether pus comes from the anterior urethra, posterior urethra, bladder, kidneys, prostate, or seminal vesicles. He properly does not attribute any importance to the two and three glass test (unless the anterior urethra has been washed out previously) in the diagnosis of posterior urethritis.

The cystoscope is considered of great importance, especially the catheterizing cystoscope.

He concludes with the following summary:

The careful localization of chronic suppurations is essential for accurate treatment. The conclusions drawn from the glass tests, as applied to suppurations of the urethra, are often misleading, while those drawn from the irrigation test are more accurate. Without cystoscopic examination, it is sometimes almost impossible to differentiate chronic suppurations of the bladder from those of the kidney. Before operating upon a suppurative lesion of the kidney, it is essential that one verify his diagnosis as to which kidney is involved, either by seeing turbid urine issuing from the corresponding ureteric orifice or by ureteral catheterization; reliance upon symptoms alone, even when these seem to leave no doubt, may lead to error.

---

BEFORE operating for pharyngeal adenoids or hypertrophied tonsils make sure that these are not merely an expression of status lymphaticus. If they are, do not employ an anesthetic. Also determine whether the patient is a hemophiliac. If he is, do not operate at all.—*American Journal of Surgery.*

### TREATMENT OF BENIGN STENOSIS OF THE PYLORUS.

In discussing the treatment of benign stenosis of the pylorus, H. L. Elsner (*Medical News*, July 22, 1905) says: "Such foods only to be taken as the dilated stomach with motor weakness can prepare to be forwarded beyond the point of resistance for further digestion. We should strive to prevent stagnation of foods with associated fermentation and the formation of organic acids; must empty the distended stomach as far as possible; prevent tetany and other nervous and distant symptoms; improve the general conditions; and treat associated symptoms as they rise by rational and natural methods, including the use of drugs, attempting by these to overcome or counteract the effect of secretory and motor anomalies."

---

### A CASE OF MENSTRUAL URTICARIA.

D. T. M. Miller (*Medical Record*, May 13, 1905) epitomizes the literature of this condition, and describes its occurrence in a girl of fifteen, who menstruates regularly and whose attacks of urticaria make their appearance seven or eight days before and cease two or three days previous to each period. Occasionally the urticaria persists until the flow begins, rarely during the first day or two of its course. During the intervals between the periods the patient is quite free from attacks, and she is perfectly healthy in other respects. The urticaria itself is of the ordinary type.

---

AT the time I had charge of the medical department of the Talladega Furnace Company and the B. & A. Railroad Company, of Alabama, I had under my observation some 1,200 men and women. My stay there continued for nineteen months, and during this time I used very little calomel, and in its stead employed Chionia for liver troubles with the best results. It is not necessary to state individual cases, but I will say that no remedy can equal Chionia as an hepatic tonic in cases of yellow skin, loss of appetite and bowel derangement. Chionia can be depended upon in clearing up these various disturbance which usually accompany or follow functional disorders of the liver.—*B. F. Laird, M. D., Covington, Ky.*

EPILEPSY OF SYPHILITIC ORIGIN.—John T. Moore, in the *Journ. of Amer. Med.* of June 10, 1905, describes such a case. Syphilitic epilepsy was suspected on account of the age of the onset. Practically all authorities agree that epilepsy coming on during adult life, unless directly traceable to injury, should be regarded as syphilitic in character. There is nothing distinctive about this form of epilepsy, though Charcot claims that the jactitations of syphilitic epilesy are of a peculiar character; viz., if the convulsions begin in the upper extremity, the lower is attacked only after the face. If the face is attacked, the convulsions spread to the upper and then to the lower extremity, and if they begin in the lower they attack the upper extremity and then the face. The convulsions never occur in the order of leg, face and arm, or arm, leg and face.

---

QUININE ADMINISTRATION OF.—Luca has compared the administration of quinine by the mouth with hypodermic injection. In each case the quantity found in the blood is very small relatively to the amount introduced into the body, and reaches its maximum after about an hour. The parasite of malaria is, however, susceptible to a minute proportion of the drug. When quinine is swallowed, more is found in both the blood and urine than when it is injected under the skin. But experience has shown that the latter method of administration is more efficacious in malaria. This is due to the prolonged action of the drug in this case, as it passes continually, though slowly, from the tissues into which it has been injected to the blood. (*Archiv. Ital. de Biol.*, March, 1905; *British Medical Journal*, July 1, 1905).

---

THERE has just been started in Paris a Hospital Clothing Association. As its title suggests the object of the society is to distribute clothing to patients who go out of the hospitals and have to look for work. The society appeals to its supporters for articles of clothing, either new or previously used, of any kind, and for money with which it may buy such articles.

---

WHEN applying a plaster dressing to the leg always include the foot if the patient is to be confined to bed; otherwise "drop foot" will develop.—*American Journal of Surgery.*

